# Electronic Cigarettes for Smoking Cessation: The Gap Between Behavior in Smokers and Medical Education

**DOI:** 10.7759/cureus.29603

**Published:** 2022-09-26

**Authors:** Michael H Bernstein, Karim Oueidat, Patrick Wasserman, Saurabh Agarwal, Grayson L Baird, Alexander Sokolovsky, Aaron Maxwell, Terrance Healey

**Affiliations:** 1 Department of Diagnostic Imaging, Rhode Island Hospital, Brown University, Providence, USA; 2 Department of Diagnostic Imaging, Brown Radiology Human Factors Lab, Providence, USA; 3 Medical School, Brown University, Providence, USA; 4 Public Health, Brown University, Providence, USA

**Keywords:** tobacco cessation, tobacco, smoking, lung cancer screen, electronic cigarettes' e-cigarettes' vaping' e-smoking

## Abstract

Introduction: E-cigarettes have engendered a great deal of controversy within the public health and medical communities.

Methods: Two cross-sectional surveys were administered. First, patients at an annual lung cancer screening appointment who self-identified as former smokers were asked about strategies for achieving and maintaining smoking cessation with open-ended questions. Second, medical students at a single university reported their opinion and knowledge of combustible cigarettes and e-cigarettes.

Results: Among the n=102 in the patient survey indicating that they used e-cigarettes or over-the-counter (OTC) nicotine replacement products for smoking cessation, 34.3% (35/102) vaped e-cigarettes, making it the second most common next to patches (47.1% {48/102}). By comparison, n=48 reported using medication. Medical student participants (n=168) were mixed regarding whether a patient should switch from traditional to electronic cigarettes (56.0% yes; 44.0% no) and reported receiving education about traditional cigarettes (92.3%) at a much higher rate than for e-cigarettes (46.4%), p<.001.

Conclusion: Many former heavy smokers undergoing a lung cancer screen used e-cigarettes to achieve smoking cessation. However, nearly half of medical students surveyed do not think patients should switch from traditional to e-cigarettes.

## Introduction

Electronic cigarettes (e-cigs) are used by just over 3% of US adults [1]. Though controversial, randomized trials suggest that e-cigs are more effective for the cessation of traditional cigarettes than nicotine replacement therapy (NRT) [2]. In one meta-analysis of nine trials where e-cigs were compared to other cessation strategies, those assigned to e-cigs had greater abstinence rates compared to people receiving NRT [2]. A more recent switching study observed that not only did e-cigs reduce NNAL concentration (a marker of tobacco use) and self-reported tobacco use at a six-week follow-up, but e-cigs also improved respiratory symptoms [3]. The results of these trials are consistent with longitudinal survey data which show that the odds of smoking cessation increase as smokers begin vaping e-cigarettes [4].

Nonetheless, some express concern that e-cig use may increase smoking rates among teens and younger adults [5], thereby counteracting a decades-long decline in tobacco utilization rates among this large demographic sector of the population [6,7]. While e-cigs are not without risk, in a position paper among 15 past presidents of the Society for Research on Nicotine and Tobacco, the authors [8] argue that e-cigs offer “potential life-saving benefits (for adult smokers}”(page e6). Despite the promise, little is known about the degree to which heavy smokers naturalistically use e-cigs as a cessation or maintenance tool. Also, since e-cigarettes are increasingly discussed during primary care visits [9], the viewpoint of future medical professionals regarding e-cigs should be investigated. We explored these topics in the current studies.

## Materials and methods

Two unique surveys were administered: one to patients and one to medical students. These are described below.

Survey 1: patients

Survey 1 was an assessment of smoking behaviors and attitudes among patients at annual lung cancer screening computed tomography (CT) appointments from 12 outpatient imaging centers in Rhode Island between 04/2019-05/2020. Patients were 55-80 year old self-identified former smokers with at least a 30-pack year history of smoking who quit in the past 15 years. Patients were asked two open-ended questions about how they achieved and maintain smoking cessation. The former was assessed with “How were you able to successfully quit smoking?” The latter was assessed with “When you do feel a craving or urge, what do you do to avoid relapsing?” This study was reviewed by the Lifespan Hospital System Internal Review Board (IRB) and deemed not to need approval.

Data Analysis

Consistent with the principles of thematic coding [10], data were content coded by a trained research assistant (author Karim Oueidat) and spot-checked by author Michael Bernstein. 

Survey 2: medical students

Survey 2 was an assessment of medical students' self-perceived competency to counsel patients on using e-cigarettes and traditional cigarettes as well as exposure to education on e-cigs and traditional cigarettes within the formal medical school curriculum. Confidence in counseling patients regarding cessation techniques was also investigated. The electronic survey was distributed by email to all 576 students enrolled at Brown University’s Alpert Medical School between 03/2020-04/2020. An IRB exemption was obtained from the Rhode Island Hospital IRB 2 Board (Providence, RI, USA).

Data Analysis

All analyses were conducted using SAS Software 9.4 (SAS Inc., Cary, NC). Demographic and yes/no questions were examined as counts and percentages. Perceptions of e-cigarettes, self-perceived ability to counsel patients on the use of e-cigarettes and adequacy of education in medical school on the topic of e-cigarettes were examined using generalized linear modeling assuming a binomial distribution. Perceptions about e-cigarettes were compared with traditional cigarettes, when applicable, using generalized mixed modeling assuming a binomial distribution with sandwich estimation. Alpha was established a priori at the 0.05 level and all interval estimates were calculated for 95% confidence.

## Results

Survey 1: patients

Among 1085 patients who completed the survey, 378 provided an open-ended response to how they achieved smoking cessation; 27.0% (102/378) reported using e-cigs or an over-the-counter (OTC) nicotine replacement product (by comparison, 12.7% {48/378} reported using medication). Of these, 34.3% (35/102) vaped e-cigarettes, making it the second most common next to patches (47.1% {48/102} (Table 1). Likewise, of the 354 participants who provided an open-ended response to how they avoid smoking during a craving or urge, 36 (10.2%) reported using e-cigs or an OTC nicotine replacement product (by comparison, 0.6% {2/354} reported using medication). Among these 36 patients, 12 (33.0%) vaped e-cigarettes, making it the second most common next to gum (52.8% {19/36}) (Table 1).

**Table 1:** Electronic cigarettes and over-the-counter nicotine replacement products for achieving and maintaining smoking cessation Denominator only reflects participants who endorsed using an e-cig or an over-the-counter nicotine product for achieving (n=102) or maintaining (n=36) smoking cessation. E-cig=electronic cigarette

	Achieve cessation	Maintain cessation
E-cigs	34.3% (35/102)	33.0% (12/36)
Patches	47.1% (48/102)	2.8% (1/36)
Gum	15.6% (16/102)	52.8% (19/36)
Lozenges	9.8% (10/102)	11.1% (4/36)

Survey 2: medical students

The survey was completed by 168 students (61% female), with a response rate of 29% (n=48-1st, 39-2nd, 30-3rd, 27-4th-year students; 24 didn’t disclose). Questions and response frequencies are detailed in Table 2. Participants were mixed in whether a patient should switch from traditional cigarettes to e-cigarettes (56.0% yes; 44.0% no). Participants reported receiving education about traditional cigarettes (92.3%) at a much higher rate than for e-cigarettes (46.4%), p<.001. Participants were less concerned about being asked their opinion on traditional cigarettes (17.9%) versus e-cigarettes (66.1%), p<.001. Relatedly, participants reported much higher confidence in counseling patients about traditional cigarettes (89.7%) compared with e-cigarettes (20.8%), p<.001. Rates believing they should receive education for traditional cigarettes (94.6%) and e-cigarettes (97.0%) failed to differ p=.21.

**Table 2:** Medical students' opinions and knowledge of e-cigarettes and traditional cigarettes

Question	Yes n (%)	No n (%)
E-Cigarette Items		
Did you have any education about e-cigarettes / vaping as part of your curriculum so far?	78 (46.43)	90 (53.57)
Are you concerned about patients asking about your opinion of e-cigarettes / vaping?	111 (66.07)	57 (33.93)
Are you confident in your ability to counsel patients on e-cigarettes / vaping?	35 (20.83)	133 (79.17)
Do you think there should be a formal educational position on e-cigarettes / vaping given to medical students?	162 (97.01)	5 (2.99)
Traditional Cigarette Items		
Did you have any education about traditional cigarettes as part of your curriculum so far?	155 (92.26)	13 (7.74)
Are you concerned about patients asking about your opinion of traditional cigarettes?	30 (17.86)	138 (82.14)
Are you confident in your ability to counsel patients on traditional cigarettes?	148 (89.7)	17 (10.3)
Do you think there should be a formal educational position on traditional cigarettes given to medical students?	159 (94.64)	9 (5.36)
Switching: Traditional cigarettes to e-cigarettes		
Your patient smokes traditional cigarettes and has expressed interest in switching to e-cigarettes / vaping but has no interest in quitting altogether. Should they switch to e-cigarettes / vaping?	93 (56.02)	73 (43.98)

## Discussion

While e-cigs are associated with worse pulmonary health compared to tobacco abstinence, research thus far suggests it is better than conventional cigarettes, [11] though there is a need for long-term follow-up studies. In our survey data, e-cigarettes were 73% as common as the more traditional cessation method of medication, equating to approximately three e-cigarette users for every four patients who took medication. Patients in the current study were referred to a lung screen by a primary care physician, and therefore constitute a group of people who interface with the medical system.

Despite the high rate of using e-cigarettes in this population, current physician trainees reported uncertainty regarding the utility of e-cigarettes for cessation. This is concerning, as a cross-sectional survey of more than 6,000 adults who were current smokers or had quit in the past two years and visited a health professional in the past one year observed that nicotine vaping products were discussed on 6.8% of visits, and was more prevalent among the US patients (8.8%) [9].

Nearly half of medical students surveyed think patients should not switch from traditional cigarettes to e-cigs. This may reflect the significantly lower rates of formal education regarding e-cigs in their training and underscores the lack of confidence in engaging with patients regarding e-cigarette use. Notably, students overwhelmingly emphasized the importance of learning about e-cigs alongside traditional cigarettes during medical education. From the medical profession standpoint, this gap should be addressed in medical school curricula. From the patient perspective, the discrepancy between the tobacco maintenance and cessation choices of real-world smokers and the training of future physicians may exacerbate uncertainties regarding the potential risks and clinical benefits of e-cigarette use.

Although public health debate about the use of e-cigs continues [12], more research is needed to evaluate the safety and efficacy of e-cigs as a smoking cessation tool in this population. To our knowledge, only one trial has examined e-cigs for tobacco use reduction among lung cancer screening patients. In this study [13], 210 smokers receiving a lung cancer screen in Italy were randomized to nicotine e-cigarettes + support, placebo e-cigarettes + support, or support alone. Although, at a 6-month follow-up, there were no group differences in abstinence rate or pulmonary health, participants in the nicotine e-cigarette group smoked fewer cigarettes per day (M=11.0, SD=6.5) compared to those in either the placebo e-cigarette (M=14.0, SD=7.9) or support alone (M=13.5, SD=6.5) group. They also had a lower exhaled carbon monoxide and nicotine dependence compared to the other two conditions. This is consistent with another trial which showed that patients with COPD who used e-cigarettes reported less smoking than COPD patients who did not use e-cigs [14]. Additional studies using the current generation of e-cigs are needed to examine whether they can reduce tobacco use in this high-risk population.

Limitations

Both surveys were conducted at only one site with modest response rates so generalizability may be limited. Smoking cessation strategies in the patient survey were only measured with two open-ended questions. Thus, e-cig use in the patient survey was not asked directly but instead coded in response to generic, open-ended questions. As a result, the prevalence rate we observed is likely an underestimation.

## Conclusions

In our patient survey, former smokers receiving a lung cancer screen were naturalistically using e-cigarettes to achieve cessation. However, future physicians lack the training and knowledge to approach vaping with their patients. Many do not believe a patient should switch from smoking to vaping. This is an important gap that should be addressed through future research and medical education.

## References

[1] Bao W, Liu B, Du Y, Snetselaar LG, Wallace RB (2020). Electronic cigarette use among young, middle-aged, and older adults in the United States in 2017 and 2018. JAMA Intern Med.

[2] Grabovac I, Oberndorfer M, Fischer J, Wiesinger W, Haider S, Dorner TE (2021). Effectiveness of electronic cigarettes in smoking cessation: a systematic review and meta-analysis. Nicotine Tob Res.

[3] Pulvers K, Nollen NL, Rice M, Schmid CH, Qu K, Benowitz NL, Ahluwalia JS (2020). Effect of pod e-cigarettes vs cigarettes on carcinogen exposure among African American and Latinx smokers: a randomized clinical trial. JAMA Netw Open.

[4] Glasser AM, Vojjala M, Cantrell J, Levy DT, Giovenco DP, Abrams D, Niaura R (2021). Patterns of e-cigarette use and subsequent cigarette smoking cessation over 2 years (2013/2014-2015/2016) in the population assessment of tobacco and health study. Nicotine Tob Res.

[5] Samet JM, Barrington-Trimis J (2021). E-cigarettes and harm reduction: an artificial controversy instead of evidence and a well-framed decision context. Am J Public Health.

[6] Miech RA, Johnston LD, O'Malley PM, Bachman JG, Schulenberg JE, Patrick ME (2022). Monitoring the Future National Survey Results on Drug Use, 1975-2020. Volume I, Secondary School Students. http://www.monitoringthefuture.org/pubs/monographs/mtf-vol1_2021.pdf.

[7] Schulenberg JE, Patrick ME, Johnston LD, O'Malley PM, Bachman JG, Miech RA (2022). Monitoring the Future National Survey Results on Drug Use, 1975-2020. Volume II, College Students & Adults Ages 19-60. Volume II, College Students & Adults Ages 19-60. Institute for Social Research.

[8] Balfour DJ, Benowitz NL, Colby SM (2021). Balancing consideration of the risks and benefits of e-cigarettes. Am J Public Health.

[9] Gravely S, Thrasher JF, Cummings KM (2019). Discussions between health professionals and smokers about nicotine vaping products: results from the 2016 ITC Four Country Smoking and Vaping Survey. Addiction.

[10] Braun V, Clarke V (2006). Using thematic analysis in psychology. Qual Res Psychol.

[11] Gugala E, Okoh CM, Ghosh S, Moczygemba LR (2022). Pulmonary health effects of electronic cigarettes: a scoping review. Health Promot Pract.

[12] Rigotti NA (2020). Randomized trials of e-cigarettes for smoking cessation. JAMA.

[13] Lucchiari C, Masiero M, Mazzocco K (2020). Benefits of e-cigarettes in smoking reduction and in pulmonary health among chronic smokers undergoing a lung cancer screening program at 6 months. Addict Behav.

[14] Polosa R, Morjaria JB, Prosperini U (2020). COPD smokers who switched to e-cigarettes: health outcomes at 5-year follow up. Ther Adv Chronic Dis.

